# Efficient Differentiation of Embryonic Stem Cells into Mesodermal Precursors by BMP, Retinoic Acid and Notch Signalling

**DOI:** 10.1371/journal.pone.0036405

**Published:** 2012-04-30

**Authors:** Josema Torres, Javier Prieto, Fabrice C. Durupt, Simon Broad, Fiona M. Watt

**Affiliations:** 1 Departamento de Biología Celular, Universidad de Valencia, Burjassot, Comunidad Valenciana, Spain; 2 Wellcome Trust Centre for Stem Cell Research, University of Cambridge, Cambridge, United Kingdom; Instituto Butantan, Brazil

## Abstract

The ability to direct differentiation of mouse embryonic stem (ES) cells into specific lineages not only provides new insights into the pathways that regulate lineage selection but also has translational applications, for example in drug discovery. We set out to develop a method of differentiating ES cells into mesodermal cells at high efficiency without first having to induce embryoid body formation. ES cells were plated on a feeder layer of PA6 cells, which have membrane-associated stromal-derived inducing activity (SDIA), the molecular basis of which is currently unknown. Stimulation of ES/PA6 co-cultures with Bone Morphogenetic Protein 4 (BMP4) both favoured self-renewal of ES cells and induced differentiation into a Desmin and Nestin double positive cell population. Combined stimulation with BMP4 and all-trans Retinoic Acid (RA) inhibited self-renewal and resulted in 90% of cells expressing Desmin and Nestin. Quantitative reverse transcription-polymerase chain reaction (qPCR) analysis confirmed that the cells were of mesodermal origin and expressed markers of mesenchymal and smooth muscle cells. BMP4 activation of a MAD-homolog (Smad)-dependent reporter in undifferentiated ES cells was attenuated by co-stimulation with RA and co-culture with PA6 cells. The Notch ligand *Jag1* was expressed in PA6 cells and inhibition of Notch signalling blocked the differentiation inducing activity of PA6 cells. Our data suggest that mesodermal differentiation is regulated by the level of Smad activity as a result of inputs from BMP4, RA and the Notch pathway.

## Introduction

Embryonic stem (ES) cells are pluripotent cells capable of differentiating into all adult cell lineages, both *in vitro* and *in vivo*. Pluripotent cells undergo symmetric self-renewal and can be maintained and expanded in cell culture indefinitely without losing their functional attributes [1]. These remarkable cells are therefore considered to be an unlimited and renewable source of adult cell types with a wide range of applications in biotechnology and biomedicine [2].

Three differentiation strategies are typically used to differentiate ES cells: aggregation of cells into embryoid bodies (EBs) in suspension; plating cells as monolayers on extracellular matrix; or co-culture with feeder cell lines that have differentiation-promoting activity [1]. One such feeder line, PA6, has been reported to have stromal-derived inducing activity (SDIA) that can direct the differentiation of ES cells into neuronal- or neural crest-derived cell types [3], [4]. Although its molecular nature is unknown, SDIA is likely to be a membrane-tethered or secreted factor(s) [5], [6], [7]. ES cells differentiated as EBs are subject to unknown and complex interactions that cannot be precisely controlled [1]. In contrast, plating pluripotent cells on feeder cells facilitates a systematic analysis of the role of signalling factors in the differentiation of ES cells.

Differentiation of pluripotent stem cells into functional cell types is driven by the coordinated activation of different signal transduction pathways, including the Bone Morphogenetic Protein (BMP) pathway [2]. BMPs are members of the Transforming Growth Factor (TGF)-β family of proteins and play an important role in regulation of stem cell fate in mammals [8]. Cellular responses to BMP are complex and context dependent, and crosstalk of BMP/MAD-homolog (Smad) signalling with Leukemia Inhibitory Factor (LIF)/Signal Transducer and Activator of Transcription 3 (Stat3), Fibroblast Growth Factor (FGF)/Extracellular Regulated Kinase (Erk) or Wingless-related MMTV integration site (Wnt)/Glycogen synthase kinase 3 beta (Gsk3-β) pathways has different cellular outcomes [9], [10]. While BMP-triggered Smad activation favours self-renewal by collaborating with LIF/Stat3 signalling in mouse ES cells, crosstalk between BMP and the Kinase insert Domain protein Receptor (Kdr, also known as Vascular endothelial growth factor receptor- 2 or Flk1) pathway induces mesoderm specification of ES cells [9], [11].

Like BMP [8], the Notch pathway plays an important role in regulating cell differentiation during development and beyond [12], [13], [14]. Activation of Notch signalling involves the engagement of Notch receptors with their cognate ligands on apposed cells and processing of the receptors by γ-Secretases and Presenilins [12], [13], [14]. BMP activation of Smads regulates Notch-dependent gene expression, causing transcriptional cooperation or antagonism depending on the gene and cell context [8], [14]. A further key pathway involved in regulating stem cell fate is Retinoic acid (RA) signalling. RA induces neural differentiation of ES cells and recent reports have implicated RA receptors in reprogramming of somatic cells to pluripotent stem cells [15], [16].

Here we present a PA6-based protocol to efficiently differentiate mouse ES cells into mesodermal cells by combined treatment with BMP4 and RA, and reveal a previously unknown contribution of Notch signalling to SDIA.

## Results

### Differentiation of ES cells with BMP4 and Retinoic Acid

**Figure 1:**
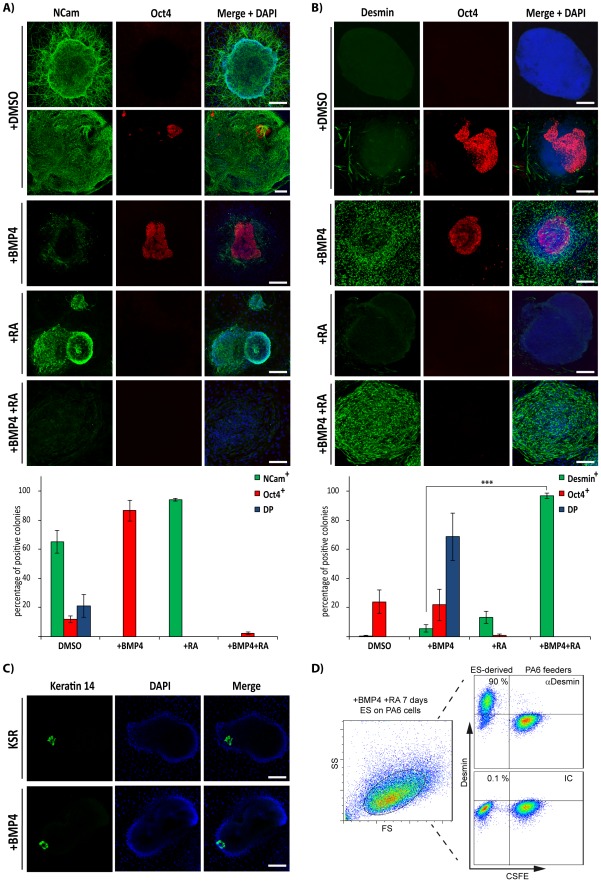
Effect of BMP4 on differentiation of ES cells cultured on PA6 feeder cells. ES cells were induced to differentiate on PA6 cells as indicated and analysed by immunofluorescence microscopy (**A, B** and **C**) or flow cytometry (**D**). The immunofluorescence images show expression of NCam and Oct4 (**A**), Desmin and Oct4 (**B**) or Keratin 14 (**C**). Quantification of the immunofluorescence experiments from (**A**) and (**B**) is shown in the lower bar diagrams. Colonies were counted as single positive for NCam (NCam+), Oct4 (Oct4+) or Double positive (DP) in (**A**); and single positive for Desmin (Des+), Oct4 (Oct4+) or double positive (DP) in (**B**). Data are represented as the average ± Standard Error of the Mean (SEM) of 3 independent differentiation experiments (***, *p*<0.001, n = 3). (**D**) ES cells were induced to differentiate for 7 days with BMP4 and RA on CSFE-labelled PA6 and Desmin expression was assessed by flow cytometry (right hand dot plots). Side (SS) and forward scatter (FS) profiles are shown in the left hand dot plot. IC: isotype control antibody. Scale bars, 50 µm.

It has been reported that ES cells cultured on PA6 stromal cells under serum free conditions are induced to differentiate into neuronal lineages, while the addition of BMP4 during the early steps of differentiation impairs neural differentiation and promotes epidermal differentiation [3]. We subjected CGR8 ES cells to the PA6-based differentiation protocol and assessed the efficiency of neuroectodermal differentiation by analysing the expression of both the pan-neural marker NCam and the pluripotency factor Oct4 (Fig. 1A). As reported [3], ES cells readily differentiated into NCam positive cells upon 9 days of differentiation under serum-free conditions, with more than 80% of colonies expressing this pan-neural marker. NCam positive cells had long neurite extensions, which are characteristic of differentiating neuroectodermal cells (Fig. 1A). We also observed clusters of Oct4 positive cells in the NCam positive colonies, with 21.0±7.7% (n = 3) of colonies being double positive (DP) for NCam and this pluripotency marker. Further, 11.8±2.3% (n = 3) of the colonies were single positive for Oct4, indicating that ES cell differentiation was not complete.

We next sought to investigate the effect of BMP4 on differentiation of ES cells co-cultured with PA6 cells. CGR8 ES cells were subjected to the PA6-based differentiation protocol, as above, and BMP4 was added from day 2 (Fig. 1A–B). Addition of BMP4 abolished expression of NCam [3] (Fig. 1A). BMP4 stimulation increased the number of Oct4 positive colonies to about 80–90% (Fig. 1B). We observed that 5.7±2.6% (n = 3) of BMP4 treated colonies were single positive for the intermediate filament protein Desmin, a marker of muscle-derived cells. Remarkably, stimulation with BMP4 led to 68±16% (n = 3) of colonies being double positive (DP) for Oct4 and Desmin. We only detected a few colonies that were positive for Desmin in the DMSO control conditions (0.6±0.8, n = 3). Our data suggest that BMP4 plays a dual role in the PA6 cell-based protocol: favouring self-renewal of ES cells and promoting their differentiation into a Desmin positive cell type.

It has been reported that early BMP4 stimulation of ES/PA6 co-cultures leads to the appearance of Keratin 14 (Krt14) positive cells after switching to serum containing medium [3], [17]. Although some cells showed Krt14 staining after 9 days of differentiation, there was no effect of BMP4 in inducing Krt14 expression (Fig. 1C).

In order to isolate and further analyse the Desmin positive cells observed in our assays, we next attempted to eliminate the undifferentiated cells. To do this, we stimulated CGR8 ES/PA6 co-cultures with all-trans Retinoic Acid (RA), which is known to induce differentiation of ES cells [15]. RA treatment of ES/PA6 co-cultures induced differentiation of ES cells into neuroectodermal cells (94±1% of NCam-positive and 10±6% Desmin-positive colonies, n = 2) and impaired expression of Oct4 (Fig. 1A and B). Combined stimulation with BMP4 and RA also eliminated Oct4 expression induced by BMP4 treatment alone (Fig. 1A and B). Remarkably, the combined action of BMP4 with RA rendered 98±2% of the colonies single positive for Desmin, compared to 6±5% with BMP4 treatment alone (*p*<0.001, n = 3) (Fig. 1B). Furthermore, flow cytometry indicated that 90% of ES cells were positive for this intermediate filament protein after 9 days of differentiation in the presence of BMP4 and RA (Fig. 1D). Similar results were obtained with the E14Tg2a ES cell line.

These results indicate that extrinsic stimulation of ES cells with BMP4 and RA together with the PA6-derived SDIA leads to highly efficient differentiation of ES cells into a cell type expressing Desmin.

### Mesodermal induction by SDIA, RA and BMP4 stimulation of ES cells

**Figure 2:**
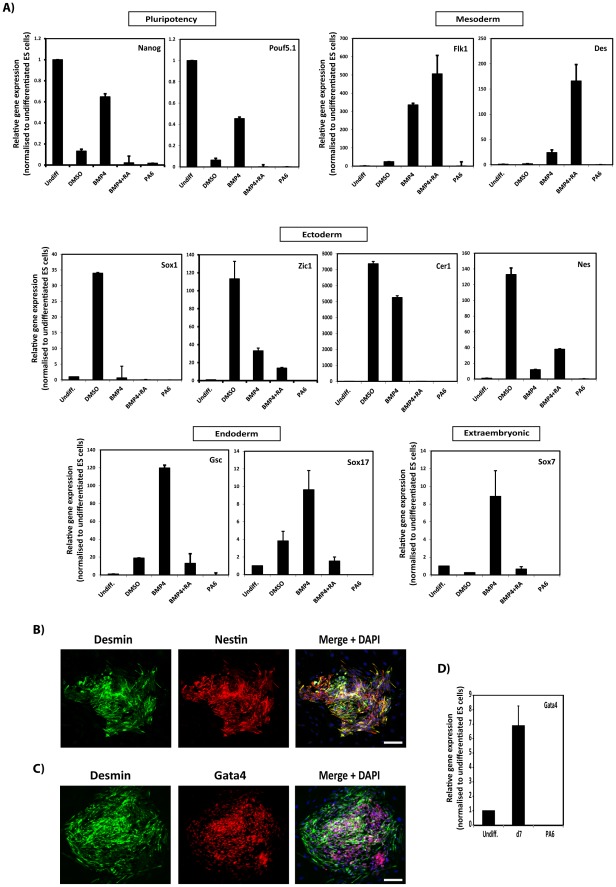
SDIA combined with BMP4 and RA treatment induces differentiation of ES cells into mesodermal precursors. (**A**) Total RNA was extracted after 9 days of ES cell culture under the conditions indicated. Expression of markers for the three germ layers was assessed by qPCR and represented as relative gene expression normalised to undifferentiated ES cells. Gene expression analyses showed robust induction of mesodermal markers by combined stimulation with BMP4 and RA. (**B–D**) ES cells were induced to differentiate on PA6 cells in serum-free medium supplemented with BMP4+Retinoic Acid (RA) for 9 days and expression of Desmin, Nestin and Gata4 was analysed by immunofluorescence staining (**B**, **C**). Images are three dimensional projections of the mean fluorescence intensity of z-stacks and show co-expression of Desmin with Nestin and Gata4. (**D**) ES cells were differentiated as in (**C**) for 7 days and expression of Gata4 analysed by qPCR as in (**A**). Undiff, undifferentiated ES cells; d7, ES cells differentiated for 7 days in the presence of BMP4 and RA; PA6, PA6 feeder cells as a negative control. Data in (**A**) and (**D**) are represented as the average ± SEM of 3 independent differentiation experiments conducted in triplicate (n = 3). Scale bars, 50 µm.

Pluripotent cells differentiate into cell lineages of the three germ layers as well as into extraembryonic tissues [1]. We investigated the embryonic origin of the cells obtained in our differentiation assays by analysing expression of markers of the three germ layers using quantitative reverse transcription-polymerase chain reaction (qPCR) (Fig. 2A). Relative to E14Tg2a undifferentiated ES cells, incubation of cells with DMSO (0.01% final concentration, as the vehicle control) reduced expression of the pluripotency genes *Nanog* and *Oct4* (also known as *Pou5f1*) to 0.18±0.07- and 0.70±0.02-fold. While treatment with BMP4 alone did not cause pronounced downregulation of *Nanog* or *Oct4*, combined stimulation with BMP4 and RA completely eliminated expression of these pluripotency markers. PA6 cells did not express *Nanog* or *Oct4*. Together with the immunofluorescence data (Fig. 1) these results indicate that combined stimulation of ES cells with BMP4 and RA suppresses expression of pluripotency markers.

E14Tg2a ES cells differentiated in the presence of DMSO showed upregulated expression of the neuroectodermal markers *Sox1*, *Nestin*, *Zic1* and *Cer1*, and did not display a pronounced increase in expression of mesodermal or endodermal makers compared to the induction of the neuroectodermal genes (Fig. 2A). Conversely, cells differentiated with BMP4 alone showed elevated expression of the extraembryonic marker *Sox7*, mesodermal markers *Flk1* and *Des*, the endodermal genes *Gsc* and *Sox17*, and the ectodermal-endodermal dual marker *Cer1* (Fig. 2A). Remarkably, cells differentiated with the combination of BMP4 and RA only displayed upregulation of the mesodermal markers *Flk1* and *Des*, suggesting that this combination of reagents induces efficient differentiation of ES cells into the mesodermal lineage. Cells differentiated with BMP4 and RA also displayed high levels of Nestin and Gata4, as observed by both qPCR and immunofluorescence analysis (Fig. 2 B–D).

**Figure 3:**
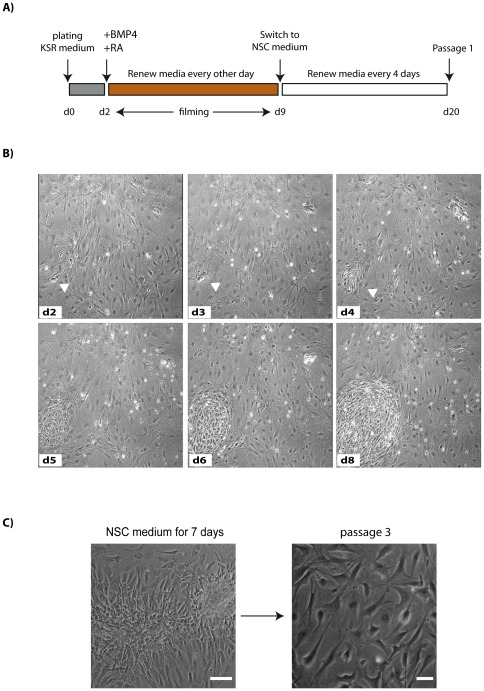
Differentiation dynamics of ES cells by BMP4+RA. (**A**) Schematic of the differentiation protocol. (**B**) Cells were recorded by time-lapse microscopy and cultured as indicated. Phase contrast images of the same field are shown. Arrowhead shows colony of ES cells that differentiated into cells of mesenchymal appearance. (**C**) Phase-contrast images of ES cell-derived mesodermal progenitors cultured for 7 days in NSC medium (left panel, scale bar 100 µm) or at the 3^rd^ passage in NSC medium (right panel, scale bar 50 µm).

To gain insight into the dynamics of CGR8 ES cell differentiation on combined stimulation with SDIA, BMP4 and RA, we monitored the co-cultures by time-lapse microscopy (Movie S1 and Fig. 3). We started recording from day 2 after plating, when BMP4 and RA were added to the medium (Fig. 3A). At this stage, ES cell colonies comprised groups of 8–12 small, epithelioid cells with scant cytoplasm, characteristic of undifferentiated ES cells (Fig. 3B, arrow head). At day 3 after plating, ES cell colonies started to lose their epithelial morphology and synchronously became fattened and spindle-shaped (Movie S1). By day 9 of differentiation, the cells showed evident mesenchymal organisation, with motile cells redistributing within the colony. However, the majority of differentiated cells stayed in close contact and did not migrate out from the colony, suggesting the presence of molecular determinants favouring cell clustering.

Cells continued to divide for the first 8 days of treatment with BMP4 and RA. However, by two weeks proliferation had ceased (data not shown), indicating that differentiation medium containing BMP4 and RA does not support growth of differentiated cells for extended periods of time.

To determine whether the differentiated cells could be serially passaged, CGR8 ES cells that had been differentiated for 9 days with BMP4 and RA were switched to serum-free medium supplemented with EGF and FGF (Fig. 3A), which are mitogens for neural stem cells (NSCs) [18]. One week after switching to NSC medium, ES-derived cells aggregated and formed tightly packed colonies, while the mitotically inactive PA6 cells began to die (Fig. 3C). Passage of the cells at this stage resulted in cultures of fibroblastic morphology (Fig. 3C) that could be serially passaged in NSC medium.

### Cells differentiated with BMP4 and RA have gene expression profiles of mesenchymal and smooth muscle cells

**Figure 4:**
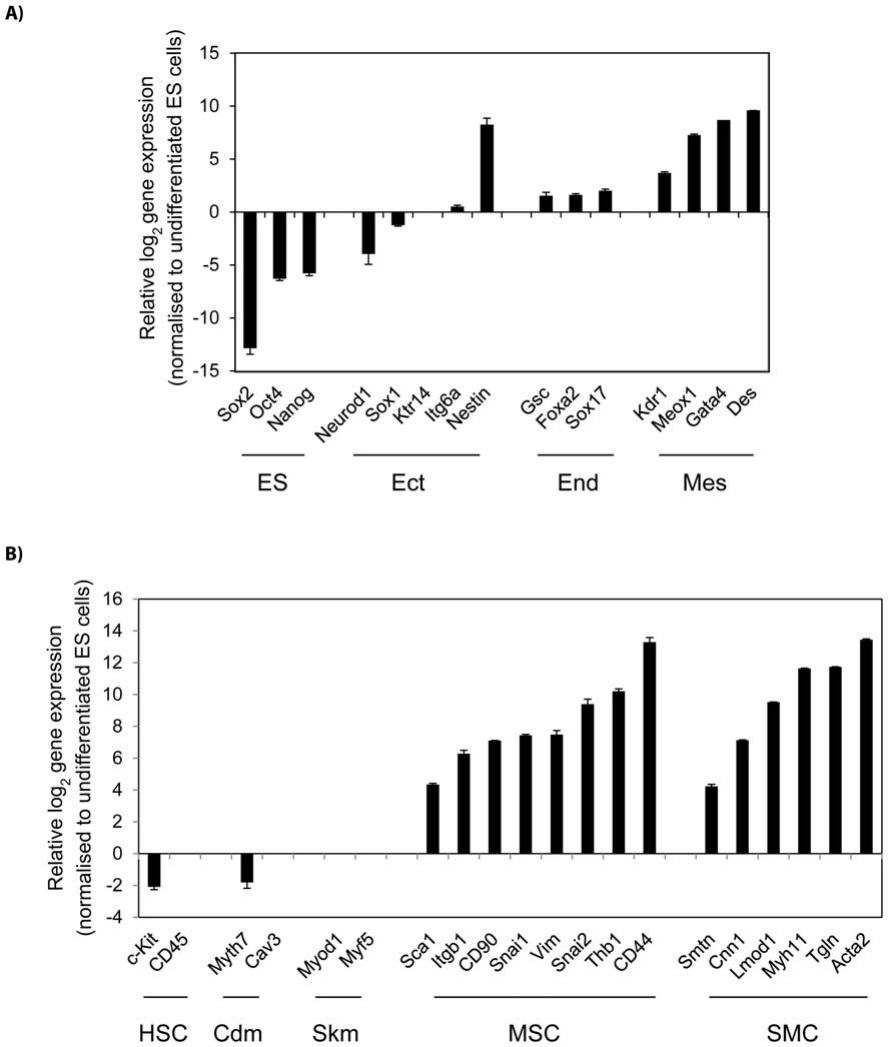
Mesenchymal gene expression signature of differentiated ES. (**A**) Marker expression profiling of ES cell-derived mesodermal precursors at the 3^rd^ passage showing downregulation of markers for undifferentiated cells and upregulated expression of mesodermal markers. (**B**) Expression of markers for mesenchymal stem (MSC) and smooth muscle (SMC) cells. Gene expression was normalised to undifferentiated ES cells and represented in log(2) scale. Data are the average ± SEM of 3 independent differentiation experiments. ES, undifferentiated ES cells; HSC, haematopoietic stem cells; Ect, ectoderm; Mes, mesoderm; End, endoderm; Cdm, cardiomyocyte; Skm, skeletal muscle.

To investigate the identity of the cells that had been passaged in NSC medium, we examined expression of markers for different cell types by qPCR. Their expression relative to undifferentiated CGR8 ES cells is shown (Fig. 4). Differentiated cells showed downregulation of the pluripotency markers *Nanog*, *Oct4* and *Sox2* (Fig. 4A). Expression of endodermal (*Gsc*, *Sox17* and *Foxa2*) and ectodermal (*Sox1*, *Neurod1* and *Ktr14*) markers was low or greatly diminished, respectively (Fig. 4A). However, cells cultured in NSC medium maintained expression of mesodermal markers (*Flk1*, *Meox1*, *Des* and *Gata4*) induced by treatment with BMP4/RA.

CGR8-derived cells cultured in NSC medium did not express markers of cardiac (*Myth7* and *Cav3*) or skeletal (*Myod1* and *Myf5*) muscle (Fig. 4B). However, we observed increased expression of *Tgln*, *Smtn*, *Myth11*, *Lmod1*, *Cnn1* and *Acta2*, genes that are characteristic of smooth muscle cells (SMC). The differentiated cells were negative for the *cKit* and *Cd45* haematopoietic stem cell markers, but showed increased expression of markers expressed in mesenchymal stem cells (MSC) (*Snai1*, *Snai2*, *Thb1*, *Vim*, *Itgb1*, *Sca1*, *Cd90* and *Cd44*) (Fig. 4B). Together, our results indicate that the differentiated cells cultured in NSC medium express markers characteristic of MSCs and SMCs.

### Modulation of BMP/Smad signalling by RA and SDIA

**Figure 5:**
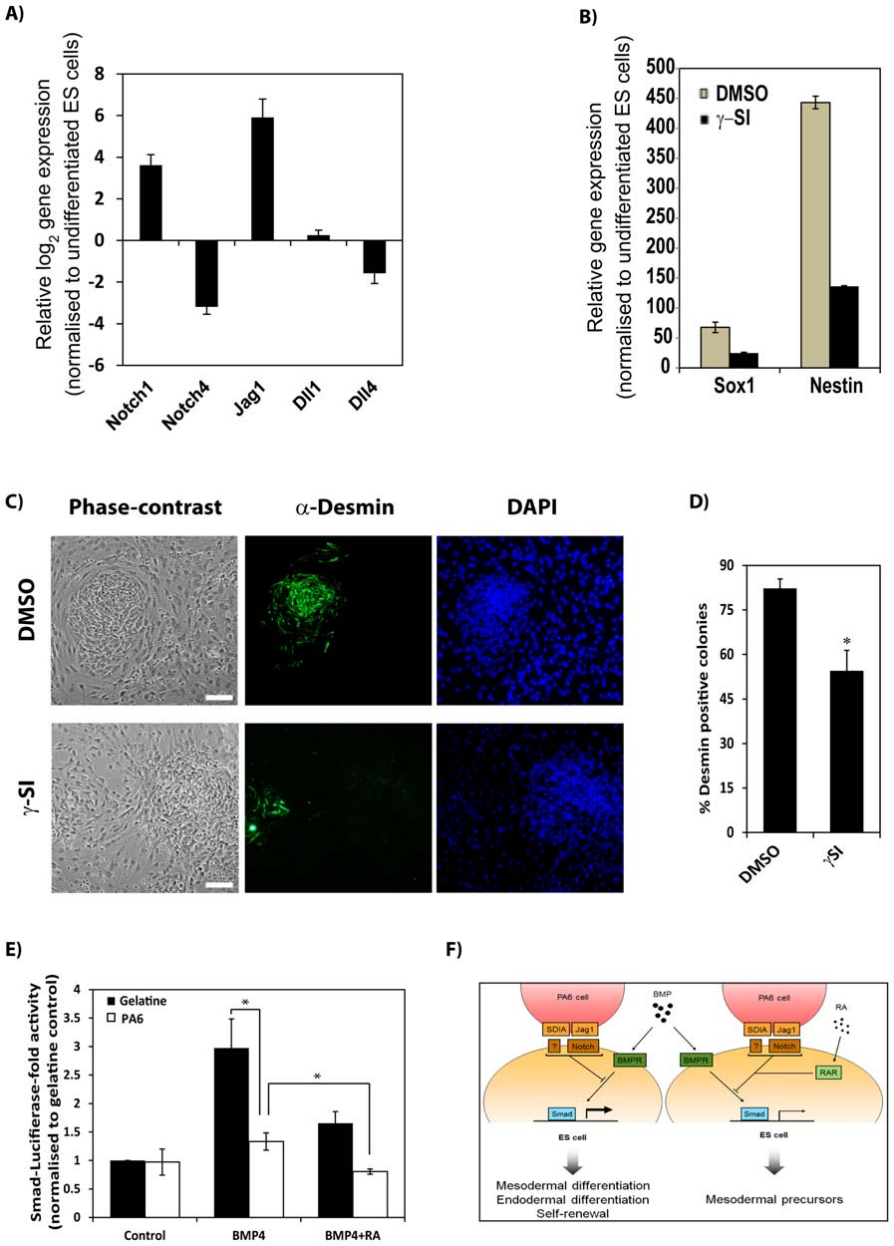
Repression of Smad-dependent transcription by RA and SDIA. (**A**) Expression of Notch ligands and receptors in PA6, determined by qPCR, relative to their values in undifferentiated ES cells. Data are represented in log(2) scale as the average ± SEM of 3 independent RNA extractions conducted in triplicate. (**B**) ES cells were induced to differentiate on PA6 cells in serum-free medium in the presence of DMSO (as vehicle control) or a γ-Secretase Inhibitor (γSI) and expression of the neroectodermal markers *Sox1* and *Nestin* was analysed by qPCR. Data are represented as the average ± SEM of a representative experiment conducted in triplicate. (**C**) ES cells were induced to differentiate on PA6 cells in serum-free medium supplemented with BMP4 and RA in the presence of DMSO (as vehicle control) or γ-Secretase Inhibitor (γSI). Representative micrographs show morphology of differentiated colonies (phase contrast; left hand panels) and expression of Desmin (green immunofluorescence; middle panels). DAPI was used as nuclear counterstaining (blue; right hand panels). (**D**) Percentage of colonies positive for Desmin, represented as the average ± SEM of 3 independent differentiation experiments. (**E**) Reporter assays of Smad-dependent transcription in ES cells plated either on gelatine (black bars) or mitomycin C-treated PA6 cells (white bars) and stimulated as indicated. Data were normalised to unstimulated (control) ES cells plated on gelatine and represented as the average ± SEM of 3 independent experiments conducted in triplicate. (*) *p*-value<0.05, n = 3. (**F**) Schematic illustrating crosstalk between the BMP4-, RA- and SDIA/Jag1-activated pathways in controlling ES cell differentiation. Scale bar in (**C**), 50 µm.

Notch signalling stimulates ES cells to undergo neuroectodermal differentiation and requires cell-cell contact [19], as does culture on PA6 cells [3]. To investigate whether SDIA acts via the Notch pathway, we first compared expression of Notch ligands and receptors in PA6 cells and undifferentiated ES cells by qPCR (Fig. 5A). Compared to undifferentiated CGR8 ES cells, PA6 were found to differentially express the Notch ligand *Jag1* and the Notch receptor *Notch1*. Inhibition of γ-Secretases inhibits both Notch activation and differentiation of ES cells into neuroectoderm [12], [14], [19]. Consistent with these earlier reports, we found that inhibition of γ-Secretases impaired expression of the neuroectodermal markers *Sox1* and *Nestin* during neural differentiation of CGR8 ES cells seeded on PA6 (Fig. 5B).

We next investigated whether Notch signalling was involved in differentiation of ES cells towards mesodermal cells. CGR8 ES cells were plated on PA6 cells with BMP4 and RA in the presence of DMSO (0.05% final concentration, as vehicle control) or a γ-Secretase inhibitor, and Desmin expression was analysed by immunofluorescence (Fig. 5C–D). Inhibition of Notch signalling significantly reduced the number of Desmin positive cells, with 54.4±6.9% of the colonies positive for this intermediate filament compared to 82.4±5% in the combined treatment with BMP4/RA (*p*<0.05, n = 3). These results suggest that activation of Notch signalling is one of the factors responsible of the differentiation-inducing attributes of PA6 stromal cells.

To test whether or not RA and SDIA directly affected BMP4 dependent signalling, we carried out luciferase assays with a canonical Smad reporter plasmid (Fig. 5E). Relative to the untreated control, BMP4 stimulation activated Smad-dependent transcription in E14Tg2a ES cells 3.0±0.5-fold and RA reduced BMP4-induced activation of the Smad reporter to 1.6±0.2-fold when the cells were plated on gelatine-coated dishes. Compared with the control cells seeded on gelatine, co-culture of ES cells with PA6 cells did not alter the basal activity of the Smad reporter in the absence of external stimuli. Activation of the Smad reporter by BMP4 was greatly impaired by co-culturing ES cells with PA6 feeders, the reporter being activated only 1.3±0.2-fold compared to 3.0±0.5%-fold when cells were cultured on gelatine-coated dishes (p<0.05, n = 3). Smad-mediated transcription was further inhibited to 0.8±0.1-fold in ES cells by addition of RA to the ES/PA6 co-cultures.

Together, these results suggest that both RA and the SDIA factor present in PA6 cells attenuate Smad-mediated transcription triggered by BMP4. Our findings further suggest that crosstalk between SDIA-, BMP4- and RA-activated signalling pathways drives the homogenous differentiation of ES cells into mesodermal progenitors by modulating BMP4 responsiveness.

## Discussion

Here we report a novel procedure to efficiently generate mesodermal progenitors from pluripotent cells in monolayer culture using the PA6 stromal cell line. Our data support a model whereby differentiation of ES cells into Desmin positive cells is controlled by cross-talk between BMP4-, RA- and SDIA-activated signalling pathways.

The PA6 stromal cell line possesses neural inductive signals that promote the differentiation of ES cells into NCam-positive neuroectodermal precursors [3]. Surprisingly, we also observed cells that were positive for the pluripotency marker Oct4 under these conditions, indicating that some ES cells escaped differentiation. In addition, BMP4 stimulation of ES/PA6 co-cultures during the early stages of differentiation favoured the expansion of undifferentiated ES cells. It has been reported that simultaneous activation of BMP4/Smad and LIF/Stat3 signalling in ES cells favours their self-renewal [9], [20]. Since PA6 cells are known to express Interleukin 6 [21], it is possible that secretion of IL-6 by PA6 cells stimulates Stat3 signalling to favour self-renewal of ES cells in the presence of BMP4. ES cells express BMP4, which allows the autocrine activation of the BMP/Smad pathway [20]. Therefore, the interaction of ES and PA6 cells can favour expansion of undifferentiated ES cells in an autocrine or paracrine manner. This would explain the presence of Oct4 positive cells in the absence of external stimuli. Undifferentiated ES cells have a very short cell cycle time and form teratomas when transplanted in vivo [1]. Thus, inhibition of BMP and/or Stat3 signalling during the first steps of ES cell differentiation could be an efficient means of inducing differentiation. In support of this, inhibition of BMP signalling with recombinant Noggin improves neuroectodermal differentiation of ES cells seeded on PA6 cells [22, JT and FMW unpublished results].

RA stimulation of ES/PA6 co-cultures induced efficient differentiation of ES cells into neuroectodermal NCam-positive cells and no Oct4-positive cells were detected [23]. It has been reported that RA stimulation induces the differentiation of ES cells into mesenchymal and skeletal muscle cells when the cells are differentiated as embryoid bodies [24], [25]. It is possible that the choice of a particular cell fate depends on both the concentration of RA and its crosstalk with different pathways. In this regard, we found that PA6 cells express the Notch ligand Jag1 and Notch signalling has been reported to steer ES cells into neuroectoderm [19]. Also, exposure of ES/PA6 co-cultures to RA has been shown to induce differentiation of ES cells into spinal cord cell progenitors [23]. It is thus possible that Notch activation in ES cells by PA6-expressed Jag1 favours their entry into the neuroectoderm lineage and RA subsequently directs these neuralised cells to spinal cord neural progenitors.

We observed that BMP4 stimulation of ES/PA6 co-cultures abrogated the expression of NCam and BMP signalling has been shown to impair neural differentiation of ES cells [26], [27]. In agreement with published work [28]–[34], the activation of this pathway promoted differentiation of ES cells into mesodermal cells. We also detected upregulation of endodermal markers upon exposure of ES/PA6 co-cultures to BMP4, suggesting that BMP4 stimulation induces endodermal differentiation. It has been reported that BMP4 collaborates with Activin A to induce mesendoderm [35], a common precursor of mesoderm and endoderm. Factors secreted by PA6 cells or differentiating ES cells, may collaborate with extrinsic BMP4 to induce endodermal differentiation.

The pleiotropic effects of stimulating ES/PA6 co-cultures with BMP alone were eliminated by combined stimulation with BMP and RA. This promoted the differentiation of ES cells into mesodermal precursors expressing Desmin. Differentiation of ES cells into mesodermal progenitors by culture on PA6 cells in the presence of BMP4 and RA was synchronous and highly efficient. Approximately 98% of colonies showed robust differentiation into Desmin and Nestin double-positive mesodermal progenitors. Nestin is expressed by multi-lineage progenitor cells [36] and it is therefore likely that the cells we have generated could be differentiated into multiple mesodermal cell types, such mesenchymal stem cells and muscle cells. Unlike EB-based differentiation strategies, our differentiation protocol proceeds in monolayer, which facilitates analysis at the cellular level, for example by time-lapse microscopy. Our protocol will be valuable for developing high throughput assays for drug discovery and/or RNAi-based screens with obvious potential biomedical applications.

The differentiated cells obtained by combined treatment of ES cells with BMP4 and RA displayed a gene expression signature resembling mesenchymal stem cells (MSCs), with high expression of mesenchymal and smooth muscle cell markers. MSCs can differentiate into multiple cell types, including chondrocytes, adipocytes and osteocytes [37]. However, we did not observe differentiation into these cell types in culture and it is possible that passaging in NSC medium suppressed further differentiation. It would be interesting to see whether the cells undergo further differentiation following transplantation *in vivo* [38].

PA6 cells expressed *Jag1* mRNA and inhibition of Notch signalling impaired ES cell differentiation into Desmin-positive cells. Notch activation regulates cell fate in ES cell-derived mesodermal progenitors [39] and it is therefore likely that Jagged1 is a key component of SDIA.

Our results support a model that underscores the role of the BMP/Smad cascade in the regulation of ES cell self-renewal and differentiation (Fig. 5F). BMP4 activation of Smad-dependent transcription in ES cells was modulated by RA or contact with PA6 stromal cells. A role for Notch activation by Jag1 in ES cell differentiation is also proposed. Notch signalling has been reported to modulate TGF-β-dependent transcription to control stem cell differentiation [40]. And recently, RA/RAR signalling was found to control the duration of BMP4-triggered phosphorylation of Smad1/5/8 [41]. Thus, cross-talk between the different pathways may provide a mechanism to finely tune Smad-dependent gene expression, thereby influencing stem cell fate.

ES cells possess a core regulatory circuitry of transcription factors that keeps them undifferentiated, yet poised to differentiate in response to extrinsic stimuli [42]. Recently, it has been shown that activated Smads are recruited to poised promoters of mesendodermal gene regulators during ES cell differentiation [43]. We suggest that RA- and SDIA- signals direct recruitment of activated Smads to a specific set of poised master genes that control the mesodermal gene expression program. The differentiation protocol we have developed provides an ideal platform for identifying those genes.

## Materials and Methods

### Cell culture, transfection and ES cell differentiation

The CGR8 ES cell line (#07032901) was from ECACC and the E14Tg2a ES cell line [44] was a gift of Professor A. G. Smith. Both ES cell lines gave similar results. ES cells were cultured on gelatinised plates in ES cell medium (Glasgow-Minimum Essential Medium (GMEM, Sigma), 10% FBS (Hyclone), 1000 units/ml ESGRO (Chemicon), 0.1 mM 2-mercaptoethanol (Sigma), 1× non-essential amino acids (Gibco) and 1× sodium pyruvate (Gibco)). Plates for ES cell culture were coated with a solution of 0.1% gelatine from porcine skin (Sigma) in PBS during 20 minutes at room temperature. PA6 cells (MC3T3-G2/PA6, RCB1127) [45] were kindly provided by Professor Y. Sasai and the RIKEN BRC through the National Bio-Resource Project of the MEXT, Japan and grown in DMEM containing 10% FBS. When indicated, PA6 were mitotically inactivated by treatment with 10 µg/ml mitomycin-C (Sigma) for 2 h at 37°C. ES cells were transfected with Lipofectamine 2000 (Invitrogen) as described [46].

Differentiation assays were conducted as described [3]. Briefly, ES cells were first cultured for two passages (4 days) in ES cell medium containing 1% FBS and 10% Knockout Serum replacement (KSR, Gibco). Then, ES cells were plated on mitomycin C-treated PA6 cells at 125 cells/cm^2^ in Differentiation medium (GMEM, 10% KSR, 0.1 mM 2-mercaptoethanol, 1× non-essential amino acids and 1× sodium pyruvate). When indicated, Differentiation medium was switched to standard Neural Stem Cell (NSC) medium (DMEM/F12 (Gibco), 2 mM L-glutamine, 0.6% glucose, 9.6 µg/ml putrescine, 6.3 ng/ml progesterone, 5.2 ng/ml sodium selenite, 25 µg/ml insulin, 0.1 mg/ml Apo-t-transferrin, 2 µg/ml heparin (sodium salt, grade II), all from Sigma), 10 ng/ml hEGF and 20 ng/ml hFGF2 (Peprotech). NSC medium was changed every 4 days. Where indicated, 1 µM all-trans retinoic acid (Sigma), 13 ng/ml BMP4 (R&D) and 4 µM γ-Secretase inhibitor (GSI, Calbiochem) were used. Time-lapse microscopy was carried out using the IncuCyte Live-Cell Imaging System (Essen Instruments).

### Luciferase assays

E14Tg2a ES cells (1.1×10^5^ cells/cm^2^) were plated on gelatinised 6-well plates the day before transfection. Cells were lipofected overnight with Renilla (0.025 µg) and Smad-Luc (0.075 µg) plasmids. The next morning, cells were trypsinised, resuspended in 2.5 ml of Differentiation medium and aliquots of 100 µl/well were added to gelatinised or PA6 cell-containing 24-well plates. 8 hours later, an equal volume of medium containing the indicated stimuli at 2× was added. The next day, cells were processed for the Dual-Glo™ Luciferase System (Promega) following the manufacturer's recommendations.

### Immunofluorescence and flow cytometry

For immunofluorescence analysis ES cells were seeded onto mitomycin C-treated PA6 plated on Poly-L-Lysine (Sigma)-coated coverslips and stimulated as indicated. Coverslips were coated with a solution of 10 µg/ml Poly-L-Lysine in distilled water for 4 hours at room temperature. Cells were then fixed for 15 min at room temperature with 4% paraformaldehyde in PBS, permeabilised for 10 minutes with 0.5% Triton-X-100 in PBS, blocked for 30 minutes with blocking buffer (3% Bovine Serum Albumin (BSA) in PBS containing 0.025% Tween-20) and incubated overnight with primary antibodies in blocking buffer. After washing with PBS supplemented with 0.025% Tween-20, cells were incubated for 1 hour with the appropriate secondary antibodies in blocking buffer containing 1 µg/ml of 4′,6′,-diamidino-2-phenylindole (DAPI) (Invitrogen), washed with PBS, mounted with Fluorsave (Calbiochem) and analysed using confocal microscopy. Confocal immunofluorescence images were taken using a LSM 510 (Carl Zeiss, Inc.) or an Olympus Fluoview FV10i (Olympus) confocal microscope equipped with 405-, 458-, 488- and 633-nm lasers. Three-dimensional reconstructions of z-stacks were performed using LSM 510 or FV10-ASW 2.1 viewer software. All images were further processed using Adobe Photoshop CS5 and compiled using Adobe Illustrator CS5.

For flow cytometry, mitomycin C-treated PA6 cells were loaded with CSFE (Molecular Probes) for 15 minutes at 37°C prior to using them as feeders for the differentiation assays. ES/PA6 co-cultures were subjected to differentiation assays as indicated. Cells were then trypsinised, washed with PBS and fixed with 4% paraformaldehyde for 10 minutes at RT. Cells were washed twice with PBS, resuspended in blocking buffer (PBS containing 2% FBS, 0.1% Saponin (Sigma)) and incubated on ice for 30 minutes. Cells were subsequently centrifuged, resuspended and incubated for 1 hour on ice in blocking buffer containing primary antibodies. Cells were washed once with PBS, incubated with secondary antibodies for 1 hour and then washed once with PBS. Labelling was measured using a FACSCanto II (BD Biosciences) equipped with 488- and 635-nm lasers and analysed using FlowJo 7.6.1 (TreeStar). At least 10,000 events from each sample were recorded.

Primary antibodies were: rabbit anti-Ncam, anti-Desmin (both from AbCam), and anti-Krt14 (Covance) diluted 1∶200, mouse anti-Oct3/4 and anti-Gata4 (both from Santa Cruz Biotechnology) diluted 1∶25, purified mouse anti-Nestin Rat-401 (DSHB) diluted 1∶100, rabbit pre-immune serum (Sigma; isotype control). Secondary antibodies were AlexaFluor^R^ 594-, 466- or 647-conjugated (Invitrogen) and were diluted 1∶1000. The monoclonal antibody Rat-401 developed by Hockfield, S was obtained from the Developmental Studies Hybridoma Bank (DSHB) developed under the auspices of the NICHD and maintained by The University of Iowa, Department of Biology, Iowa City, IA 52242.

### RNA isolation and qPCR analysis

Total RNA was extracted using TRIzol reagent and cDNA synthesised using SuperScript III reverse transcriptase kit (both from Invitrogen). cDNA products were amplified using an Applied Biosystems StepOne plus Fast Real-Time PCR system. Taqman probes were from Applied Biosystems.

### Statistical analysis

All values are represented as means ± SEM. For each experiment, the number of independent assays is indicated as “n”. Differences among means were calculated by the two-way Student's t-test. For relative values, the *arcsin* transformation was applied to the data to obtain normally distributed values. Significance was set at: *p<0.05, **p<0.01, ***p<0.001. Representations and statistical analysis were carried out using *Microsoft Excel 2010* and the *Statistical Package for the Social Sciences* (SPSS) software.

## Supporting Information

Movie S1 **Differentiation of ES cell by BMP4+RA.** ES cells were subjected to differentiation as illustrated in Fig. 3 and recorded by time-lapse microscopy from day 2 of differentiation. Time 0 indicated in the movie frame corresponds to day 2 of the experiment. The time course experiment points to day 3–4 as the onset of differentiation.
(MOV)
